# Efficient and Stable Perovskite Solar Cells Based on Inorganic Hole Transport Materials

**DOI:** 10.3390/nano12010112

**Published:** 2021-12-30

**Authors:** Helen Hejin Park

**Affiliations:** Advanced Materials Division, Korea Research Institute of Chemical Technology (KRICT), Daejeon 34114, Korea; hhpark@krict.re.kr

**Keywords:** metal halide perovskite, solar cell materials, hole transport layers, photovoltaics

## Abstract

Although power conversion efficiencies of organic-inorganic lead halide perovskite solar cells (PSCs) are approaching those of single-crystal silicon solar cells, the working device stability due to internal and external factors, such as light, temperature, and moisture, is still a key issue to address. The current world-record efficiency of PSCs is based on organic hole transport materials, which are usually susceptible to degradation from heat and diffusion of dopants. A simple solution would be to replace the generally used organic hole transport layers (HTLs) with a more stable inorganic material. This review article summarizes recent contributions of inorganic hole transport materials to PSC development, focusing on aspects of device performance and long-term stability. Future research directions of inorganic HTLs in the progress of PSC research and challenges still remaining will also be discussed.

## 1. Introduction

Photovoltaic (PV) technology research has been mainly focused on high efficiencies with low-cost materials and fabrication processes, but the high stability of working devices is also crucial for commercialization. Perovskite solar cells (PSCs) have impressively increased their unit-cell efficiency from 3.8% to 25.5% within about a decade [1], approaching single-crystal silicon solar cell efficiency values. Organometallic halide perovskites are based on the chemical formula of *AMX*_3_, where *A* is organic or metal cations, such as formamidinium ((NH_2_)_2_CH^+^ (FA^+^)), methylammonium (CH_3_NH_3_^+^ (MA^+^)), or Cs^+^, *M* is metal ions such as Pb^2+^ or Sn^2+^, and *X* is halogen ions, such as I^−^, Br^−^, or Cl^−^. Organometallic halide perovskites exhibit features of an ideal light absorber material, including high absorption coefficients (~10^−4^ cm^−1^), long carrier diffusion lengths (>1 μm), ambipolar charge transport capabilities, and low exciton binding energy (20–50 meV) [2].

Despite the remarkable growth in efficiency enhancement, compared to silicon solar cells, the working device stability still needs improvement. Degradation in PSCs can occur from both internal and external factors. Internal degradation factors include ion migration in the perovskite, lattice relaxation at interfaces, and diffusion of HTL dopants, while external degradation factors include exposure to light, heat, bias, moisture, and oxygen [3].

Perovskite solar cell device structures normally consist of the perovskite layer in between an HTL and electron transport layer (ETL), with a transparent conducting oxide (TCO), such as fluorine-doped tin oxide (FTO) or indium tin oxide (ITO), on top of a glass substrate and a metal, such as silver (Ag), gold (Au), or aluminum (Al), as the top contact [4]. Depending on the sequence of the transport layer type, the device structure is called *n*-*i*-*p* for the *n*-type ETL on the bottom (on top of the TCO) and the *p*-type HTL on top of the light-absorbing perovskite layer (below the top metal contact), and *p*-*i*-*n* for the HTL on the bottom and ETL on top of the light-absorbing layer.

The conventional HTL is based on organic materials, which are susceptible to elevated temperatures, and diffusion of HTL dopants into the perovskite layer can also cause degradation of PSC devices. Conventional *n*-*i*-*p* structures usually use poly[bis(4-phenyl)(2,5,6-trimethylphenyl)amine] (PTAA) and 2,2′,7,7′-tetrakis-(*N*,*N*-di-4-methoxyphenylamino)-9,9′-spirobifluorene (spiro-OMeTAD) as the HTL [5,6], whereas conventional *p*-*i*-*n* structures normally use poly(3,4-ethylenedioxythiophene) polystyrene sulfonate (PEDOT:PSS) and PTAA [7,8,9]. Both spiro-OMeTAD and PTAA normally have dopants, such as lithium bis(trifluoromethanesulfonyl)imide and 4-tert-butylpyridine, to improve the conductivity of the HTLs. However, such dopants easily migrate out of the HTL diffusing into the perovskite layer, which leads to degrading the PSCs. PEDOT: PSS-based PSCs are known to suffer from stability issues due to the hygroscopic and acidic characteristics of PEDOT:PSS.

Suitable HTL materials should (i) possess suitable band alignment with the light absorber layer for effective transfer of the photoexcited holes from the perovskite to the HTL, (ii) have high carrier mobility to enhance the fill factor by reducing series resistance, (iii) exhibit a wide optical bandgap so that there will be no contribution as a second light absorber layer, (iv) possess high transparency to minimize optical losses, (v) show adequate hydrophobic nature to tolerate long-term exposure to humidity, (vi) have low materials and fabrication costs, (vii) be environmentally friendly, and (viii) show good stability against light and heat [10,11,12].

While much effort has been made to modify the perovskite layer to enhance device stability [13,14,15,16], another straightforward approach to enhance the working stability of PSCs would be to replace these organic HTLs with inorganic materials. In this review, the recent progress of inorganic HTLs in PSCs is summarized based on device performance and stability in Section 2. Inorganic HTL materials covered include nickel oxide (NiO_x_), copper thiocyanate (CuSCN), Cu-based delafossite materials, such as CuAlO_2_, CuCrO_2_, CuGaO_2_, and CuFeO_2_, copper oxide (CuO_x_), copper iodide (CuI), copper sulfide (CuS), cobalt oxide (CoO_x_), chromium oxide (CrO_x_), molybdenum oxide (MoO_x_), vanadium oxide (VO_x_). Section 2 will also discuss the long-term stability of the inorganic HTL-based PSCs. Progress in the photovoltaic performance and device stability are summarized in Table 1 and Table 2, respectively. Conclusions along with future directions will be discussed in Section 3.

## 2. Device Performance and Stability of Inorganic Hole Transport Materials-Based PSCs

### 2.1. Nickel Oxide

Nickel oxide has already been used in dye-sensitized solar cells (DSSCs) and organic photovoltaics (OPV) as the *p*-type HTL before being applied in PSCs [62,63]. NiO_x_ exhibits high transparency from its wide bandgap (3.6 eV), deep valence band (−5.2 to −5.4 eV), and high carrier mobility (0.1 cm^2^/Vs) while having suitable stability against light, heat, and moisture, making it a suitable HTL candidate for PSCs [64]. NiO_x_ has been applied to PSCs in both *n*-*i*-*p* and *p*-*i*-*n* structures. There are more considerations required when applying NiO_x_ on top of the perovskite layer in *n*-*i*-*p* structures, as sputtering, water, or polar organic solvents can damage the underlying perovskite layer. NiO_x_ was applied in *n*-*i*-*p* structured PSCs by dc magnetron sputtering [17]. The efficiency was only 7.3%, but the unencapsulated device remained stable for 60 days in a 25 °C ambient atmosphere with 28 ± 2% relative humidity without light soaking. Chlorobenzene-dispersed NiO_x_ HTL can also be directly deposited on the perovskite films without decomposing the perovskite, resulting in efficiencies over 9% [18].

**Figure 1:**
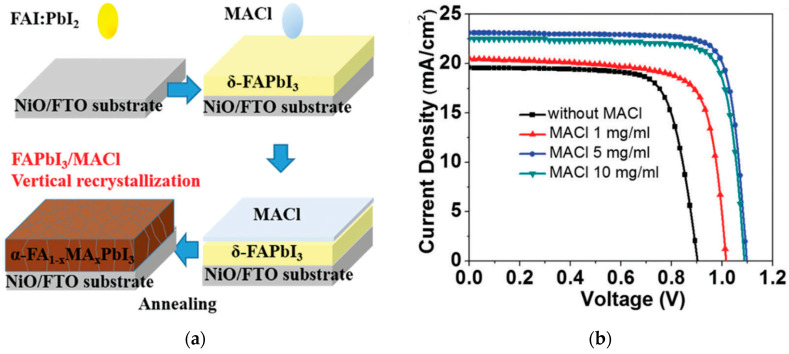

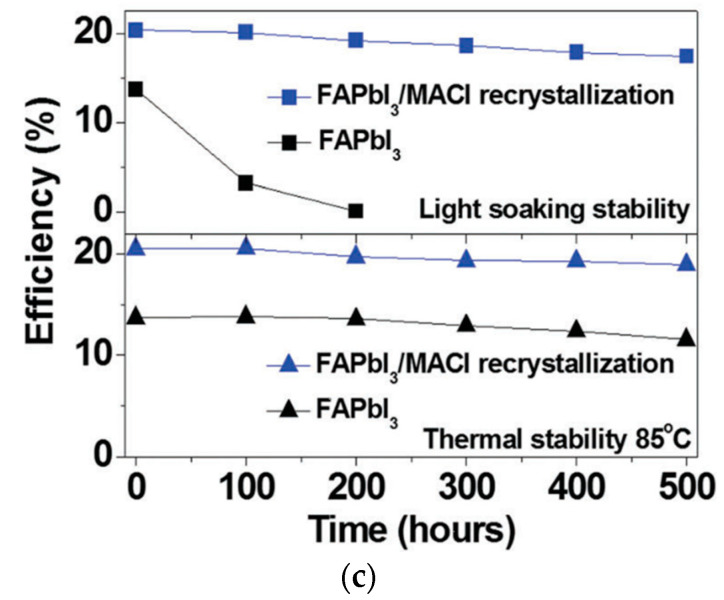
NiO_x_-incorporated PSCs: (**a**) Illustration of vertical recrystallization method. (**b**) Illuminated current density-voltage (*J*-*V*) scans of perovskite with various MACl treatments. (**c**) Stability comparison of pristine and MACl-treated FAPbI_3_ under 1 SUN light soaking at maximum power point tracking (MPPT) at ~25 °C or 85 °C. Reproduced from the work of [21] with permission from the Royal Society of Chemistry, 2017.

**Figure 2:**
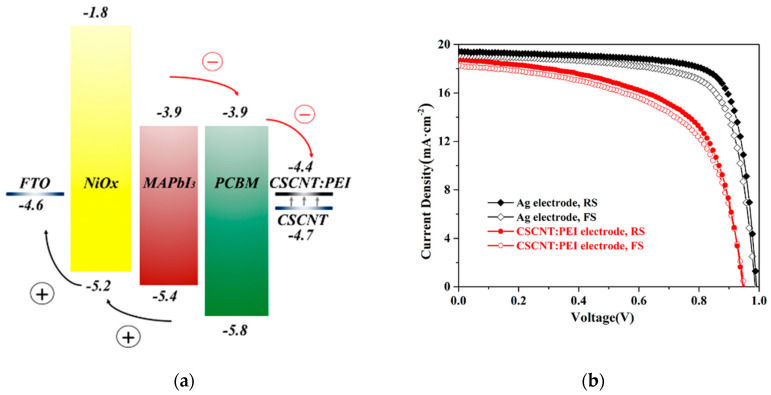

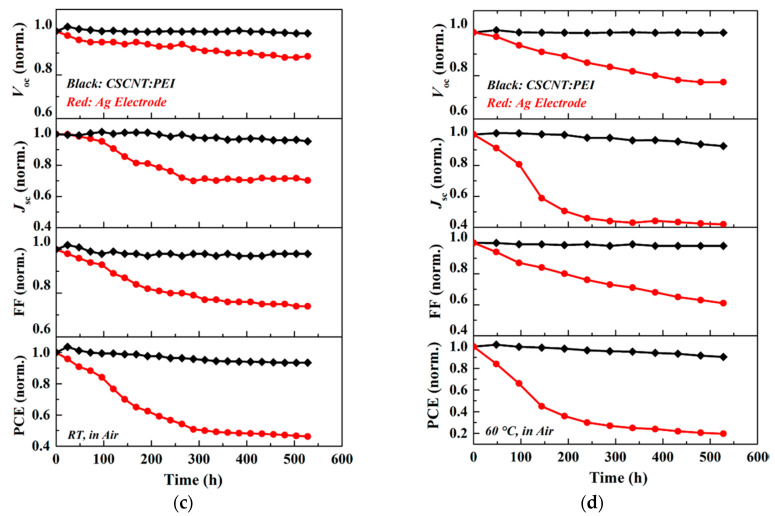
NiO_x_-incorporated PSCs: (**a**) Schematic illustration of the band alignment and transfer process. (**b**) Illuminated *J*-*V* scans of PSCs with different electrodes. (**c**) Evolution of average photovoltaic performance parameters as a function of time at room temperature and (**d**) at a constant temperature of 60 °C. Reproduced from the work of [60], with permission from the American Chemical Society, 2018.

**Figure 3:**
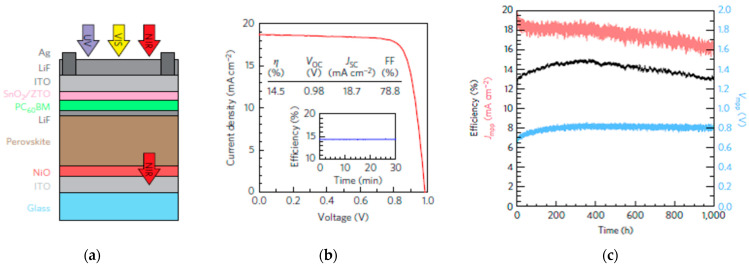
NiO_x_-incorporated PSC: (**a**) Schematic illustration of the device structure. (**b**) Illuminated *J*-*V* scan of the champion device. Efficiency at the maximum power point (inset). (**c**) Efficiency (black), current density (*J_MPP_*, red), and voltage (*V_MPP_*, blue) for a single-junction PSC device with no encapsulation for 1000 h of MPPT. Reproduced from the work of [61], with permission from the Nature Publishing Group, 2017.

For *p*-*i*-*n* configurations, power conversion efficiencies (PCEs) of 17.3% [19] and 17.6% [20] were achieved by pulsed laser deposition, by controlling the deposition time and oxygen partial pressure, and by solution processing, respectively. Vertical recrystallization of the perovskite layer and co-doping the NiO_x_ with lithium (Li) and magnesium (Mg) resulted in a PCE over 20%, and the device stability maintained over 85% of its initial PCE under maximum power point tracking (MPPT) conditions for over 500 h, as shown in Figure 1 [21]. Li-doping increases the *p*-type conductivity, whereas Mg-doping adjusts the valence band energy level. Previous reports demonstrate NiO_x_-based PSCs maintaining 85% of its initial PCE at 60% humidity and 60 °C for 500 h for encapsulated devices (Figure 2) [60], and 100% of its initial PCE under 1 SUN at 40% humidity and 35 °C for 1000 h under MPPT conditions for devices without encapsulation (Figure 3) [61].

**Figure 4:**
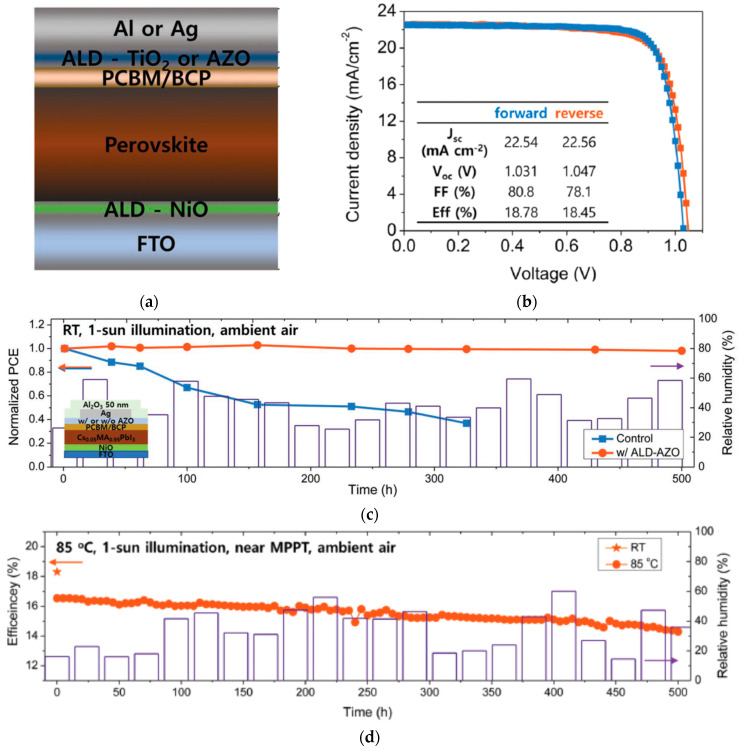
NiO_x_-incorporated PSC: (**a**) Schematic illustration of the device structure. (**b**) Illuminated *J*-*V* scans of the champion device with various ETLs. (**c**) Efficiency evolution over 500 h for devices passivated with 50 nm of Al_2_O_3_ under continuous 1 SUN illumination with a 420 nm cutoff UV filter at room temperature in ambient air (20–60% relative humidity). (**d**) Efficiency evolution over 500 h for PSC with AZO and 50 nm of Al_2_O_3_ near the MPPT under 1 SUN illumination with a 420 nm cutoff UV filter at 85 °C in ambient air. Reproduced from [23], with permission from Wiley, 2018.

Application of NiO_x_ by atmospheric pressure spatial atomic layer deposition (s-ALD) system, a more rapid method than conventional ALD [65,66], in *p*-*i*-*n* structured PSCs has been demonstrated. Employing high-quality and high-uniformity NiO_x_ HTLs in PSCs resulted in PCEs over 17% with negligible hysteresis and fill factors over 80% [22,67]. Perovskite films with improved efficient collection of charge carriers and intrinsic electronic quality were enabled from the high uniformity of NiO_x_, resulting in PSC devices with reduced interfacial trapping and improved open-circuit voltage (*V_OC_*). NiO has also been applied to *p*-*i*-*n* PSCs using plasma-enhanced ALD (PEALD), resulting in PSCs over 17% [24]. For *p*-*i*-*n* structured PSCs, atomic layer deposition (ALD) of NiO employed in PSCs resulted in efficiencies of over 18% [23]. The ALD NiO-based PSCs maintained over 99% of their initial PCE at room temperature conditions and 87% at 85 °C under 1 SUN, MPPT, as shown in Figure 4.

Researchers have found various methods to dope NiO_x_ to improve conductivity, and thus, the PSC device performance. Copper-doped NiO_x_ (Cu:NiO_x_) resulted in efficiencies over 17.8% using a low-temperature combustion process, outperforming the conventional sol-gel-derived high-temperature Cu:NiO_x_ PCE of 15.5% [25]. Despite the reduced process temperature Cu:NiO_x_ prepared by this combustion process has a tendency to be better than the conventional high-temperature sol-gel process in terms of optical transparency, crystallinity, and electrical conductivity. Yue et al. further improved Cu:NiO_x_-based PSCs by doping the methylammonium lead halide perovskite with chlorine to improve the open-circuit voltage, modifying the aluminum cathode with zirconium acetylacetonate (Zracac), and employing fluorine-doped tin oxide (FTO), which resulted in a PCE of 20.5% [26]. Doping of NiO_x_ with lithium (Li) and magnesium (Mg) in PSC has also been demonstrated, resulting in PCEs of 18% [28] and 18.5% [30], respectively. Li-doped NiO surface by a hot-casting method enabled highly crystalline MAPbI_3_, resulting in hysteresis-free, efficient, and photostable PSCs. Limitations of poor fill factor and short-circuit current density in sputtered NiO_x_-based PSCs can be overcome through introducing Mg at a low oxygen partial pressure deposition condition. Doping NiO_x_ with Li and Mg have also demonstrated devices with PCEs of above 20.7% with suitable stability for 500 h in a light soaking and thermal aging test [21], and 19.2% with retaining >80% of the initial PCE after light soaking for 1000 h or thermal exposure at 85 °C for 500 h [27]. Doping NiO_x_ with cesium (Cs) exhibits higher conductivity and higher working function, resulting in improved PCE from 16.0% to 19.4% when applied in PSCs [29]. Doping with cobalt (Co) improved PSC devices from efficiencies of 16.0% to 17.8% due to less charge accumulation and open-circuit voltage loss from the improved hole mobility and reduction in interfacial resistance [31]. Thus, NiO_x_ can be doped by various elements, such as Cu, Li, Mg, Cs, and Co, to improve the conductivity and enhance the efficiency of the PSC by reducing interfacial resistance at the HTL/perovskite interface.

### 2.2. Copper Thiocyanate

Copper thiocyanate (CuSCN) has a wide bandgap of 3.9 eV and appropriate valence band energy level of −5.3 eV with carrier mobility of 10*^−^*^2^–10*^−^*^1^ cm^2^/Vs and superior thermal stability compared to spiro-OMeTAD, making it a suitable HTL candidate for PSCs [68]. Furthermore, it is solution processable with a low cost, showing potential in commercialization. CuSCN has been applied to PSCs in mostly *n-i-p* configurations. Madhavan et al. demonstrated PCE values of 16.6% from a thick CuSCN layer of 500 nm fabricated by doctor blading, whereas spin-coated CuSCN films of 30 nm resulted in PSCs of 15.4% [32]. Jung et al. demonstrated CuSCN-based PSCs with a PCE of 18.0% using a formamidinium-based lead halide perovskite, (FAPbI_3_)_0.85_(MAPbBr_3_)_0.15_, which is more tolerant to thermal stress than MAPbI_3_ [33]. CuSCN-based PSCs retained 60% of the initial PCE in air at 125 °C with 40% relative humidity for 2 h, while spiro-OMeTAD-based PSCs retained only 25% of their initial PCE under the same conditions.

**Figure 5:**
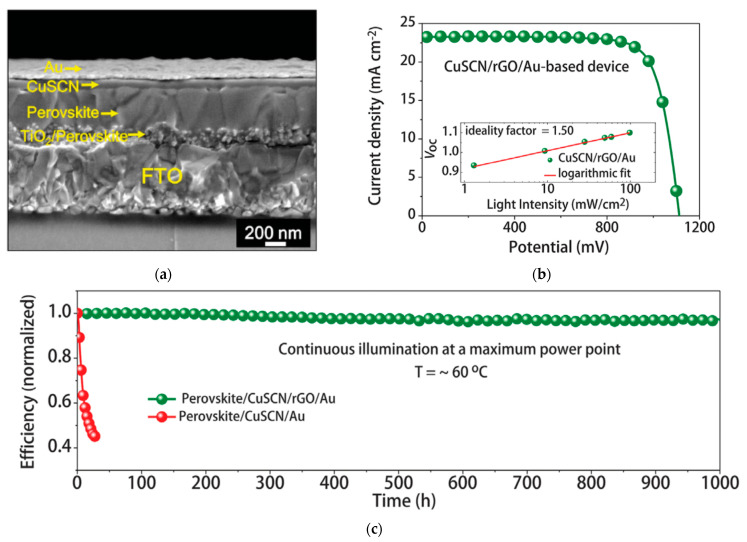
CuSCN-incorporated PSC: (**a**) Cross-sectional scanning electron microscopy (SEM) of the PSC device stack. (**b**) Illuminated *J*-*V* scan of the champion device. The *V_OC_* vs. illumination intensity with an ideality factor of 1.50 (inset). (**c**) Efficiency evolution of unencapsulated devices at MPPT for 1000 h under 1 SUN at 60 °C in nitrogen. Reproduced from the work of [34], with permission from the American Association for the Advancement of Science, 2017.

Highly conformal CuSCN layers were formed through a fast solvent removal method, facilitating rapid carrier extraction and collection, resulting in a PCE of 20.4% [34]. Applying a reduced graphene oxide (RGO) layer before the top Au contact further enhances the stability by reducing potential-induced degradation from the reaction of Au and SCN^−^ anions at the CuSCN/Au contact. These PSCs showed excellent thermal stability under long-term heating. Over 95% of the initial PCE is maintained under 1 SUN, 60 °C, in nitrogen (N_2_) atmosphere with MPPT conditions after 1000 h surpassing the light stability of devices based on spiro-OMeTAD, as shown in Figure 5. Light and heat stability of CuSCN-based PSCs can be greatly enhanced by the insertion of an RGO layer in between CuSCN and Au to block anion diffusion.

### 2.3. Copper Iodide

**Figure 6:**
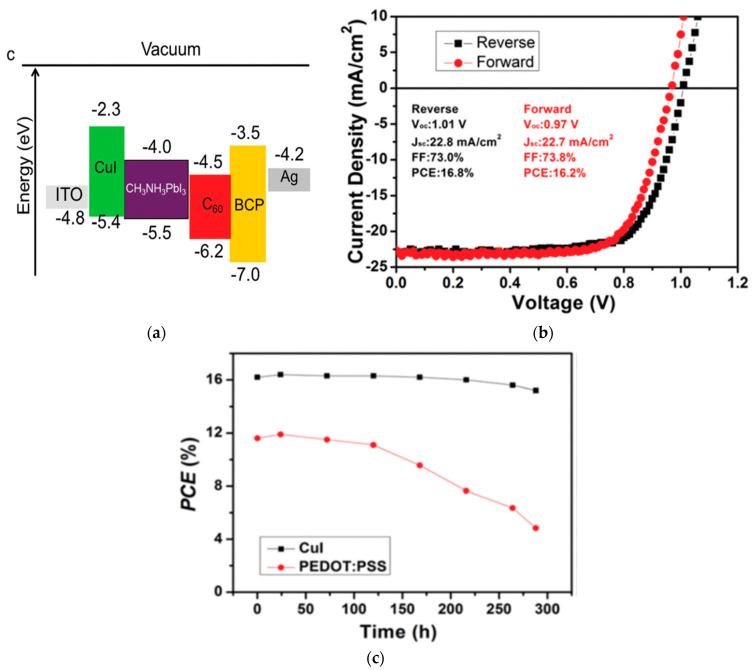
CuI-incorporated PSC: (**a**) Energy level diagram of each layer in the PSC. (**b**) Illuminated *J*-*V* scan of the champion device. (**c**) Efficiency evolution of unencapsulated devices in ambient atmosphere. Reproduced from the work of [36], with permission from the Royal Society of Chemistry, 2016.

Copper iodide (CuI) is a *p*-type semiconductor with a valence band energy level of −5.2 eV, a large bandgap of 3.1 eV, and hole mobility of 0.5–2 cm^2^/Vs [69]. Due to its hydrophobicity, CuI shows suitable ambient stability compared to PEDOT:PSS. CuI has already been widely used in OPV and DSSCs as an HTL and is a promising alternative in terms of low-cost and large-scale industrial commercialization [70]. CuI has been applied to PSCs in both *p-i-n* and *n-i-p* configurations, with PCEs of 7.5% [35] and 16.8% [36], respectively. CuI in the *n-i-p* structured device was successfully applied in a planar structured device and displayed significantly reduced hysteresis compared to the conventional devices based on spiro-OMeTAD. CuI in a *p-i-n* structured device maintained 93% of its initial PCE after storage in 25% humidity at room temperature without illumination for 300 h, showing better air stability than the reference PSC based on PEDOT:PSS, as shown in Figure 6 [36]. Ramachandran et al. reported a two-step electrodeposition method of preparing CuSCN on top of CuI on ITO with a carbon back electrode, resulting in a PCE of 20.4% [37].

### 2.4. Copper Oxide

**Figure 7:**
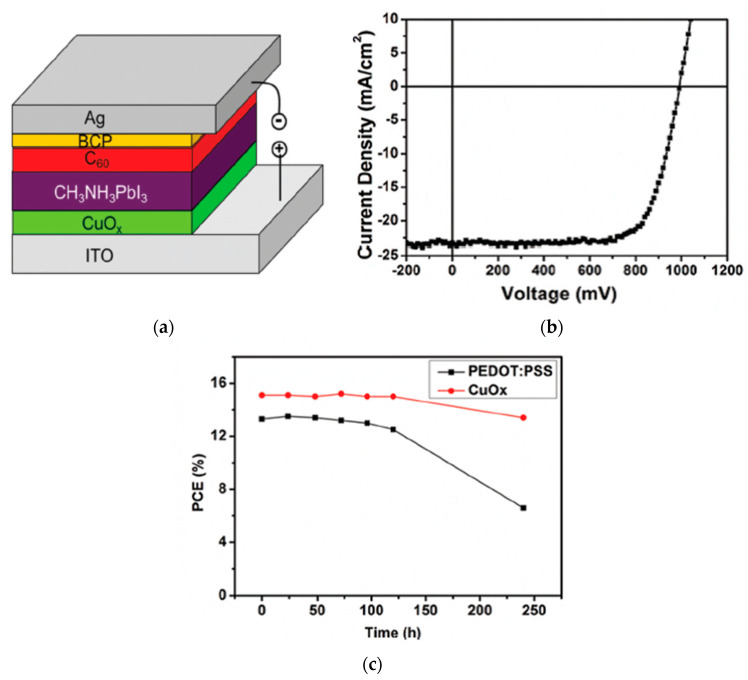
CuO_x_-incorporated PSC: (**a**) Schematic illustration of the PSC device structure. (**b**) Illuminated *J*-*V* scan of the champion device. (**c**) Efficiency evolution of unencapsulated devices in ambient atmosphere. Reproduced from the work of [38], with permission from the Royal Society of Chemistry, 2016.

Copper oxides, such as cuprous oxide (Cu_2_O) and cupric oxide (CuO), are *p*-type semiconductors composed of environmentally friendly and abundant elements with low cost and suitable heat and ambient stability [71]. CuO has a bandgap of 1.3 eV and a valence band energy level of approximately −5.4 eV, while Cu_2_O has a bandgap of 2.1 eV, valence band energy level of −5.3 to −5.4 eV, and high carrier mobility of ~100 cm^2^/Vs [72]. Solution-processed CuO_x_ has been applied to *p*-*i*-*n* PSC devices, exhibiting high transparency in the visible region and a smooth surface, resulting in a PCE of 17.1% [38]. Approximately 90% of the initial PCE was maintained after storage in air without encapsulation for 300 h, showing enhanced air stability compared to the conventional PSC device based on PEDOT:PSS, as shown in Figure 7. Efficiencies were further improved to 17.4% by applying solution-processed CuO_x_, which exhibit high optical transmittance, high work function, and excellent hole-extracting ability [40]. Conventional PEDOT:PSS-based PSC devices resulted in 12.0% efficiency. The high work function of CuO_x_ enables ohmic contact at the perovskite/CuO_x_ interface, which reduces open-circuit voltage loss.

**Figure 8:**
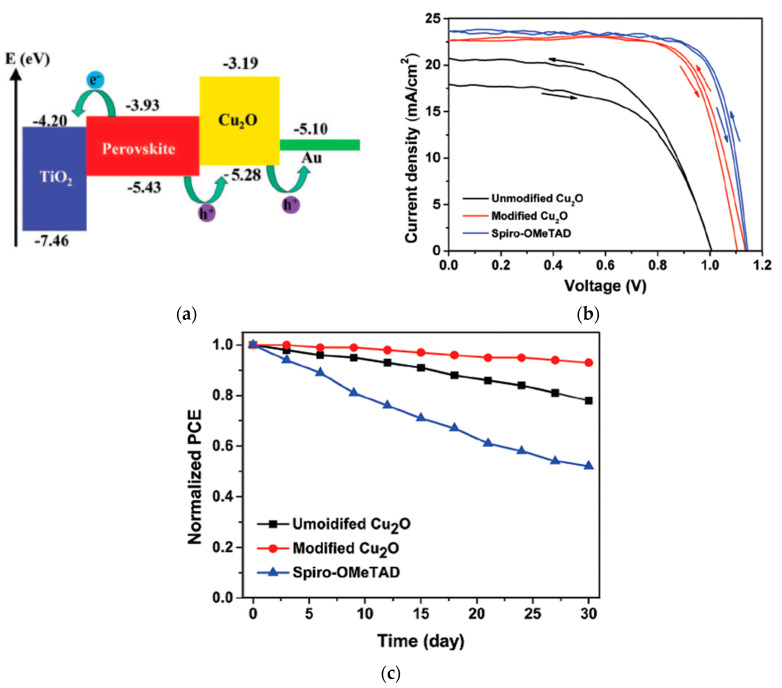
Cu_2_O-incorporated PSC: (**a**) Schematic illustration of the band alignment and carrier extraction. (**b**) Illuminated *J*-*V* scans of the PSC with various HTLs. (**c**) Efficiency evolution of unencapsulated devices in ambient atmosphere for over 30 days. Reproduced from the work of [41], with permission from Wiley, 2019.

**Figure 9:**
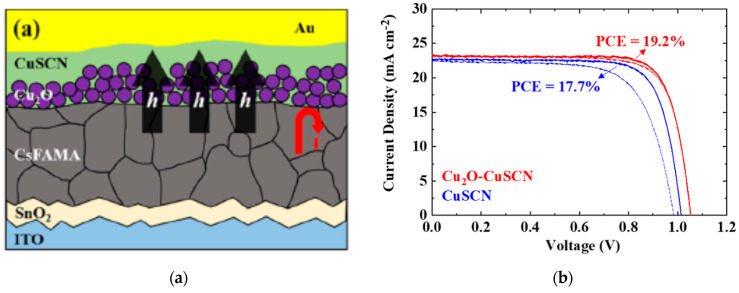

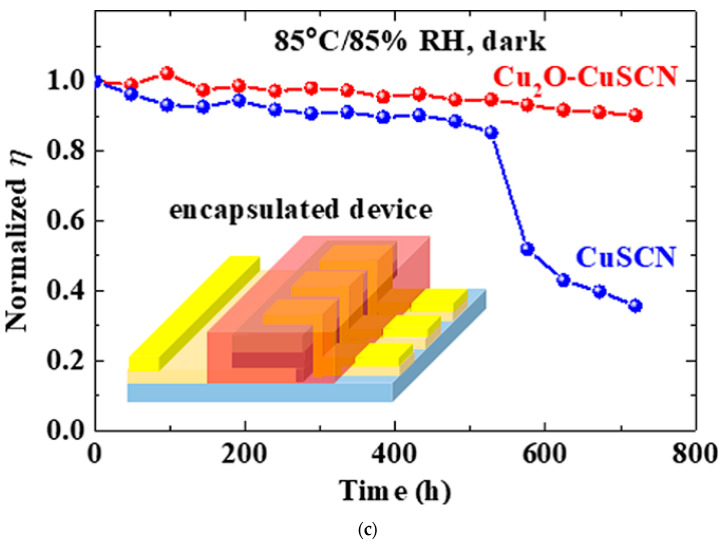
Cu_2_O-CuSCN-incorporated PSC: (**a**) Schematic illustration of the device architecture. (**b**) Illuminated *J*-*V* scans of the PSC with various HTLs. (**c**) Efficiency evolution of encapsulated devices for over 720 h at 85 °C, 85% relative humidity. Reproduced from the work of [42], with permission from the American Chemical Society, 2020.

Further improvements in CuO_x_-based PSCs were made by Rao et al. by Cl doping of the perovskite layer based on a modified one-step fast deposition-crystallization method leading to a PCE of 19.0% [39]. Cl-doping MAPbI_3_ perovskite films remarkably improves the perovskite hole mobility and film morphology, greatly increasing the device recombination resistance and reducing the intrinsic defects. Quantum dot (QD) Cu_2_O dispersed in a nonpolar solvent has been spin-coated on top of the perovskite layer in a mesoporous *n*-*i*-*p* structure, resulting in a PCE of 18.9% [41]. Surface modification of Cu_2_O allows direct deposition on the perovskite film without decomposing the perovskite, resulting in a significantly higher PCE compared to the unmodified Cu_2_O, which resulted in a PCE of 11.9%. The dopant-free method and hydrophobic surface of Cu_2_O enable excellent long-term stability maintaining over 90% of the initial PCE for over 1 month when stored in air without encapsulation with a relative humidity of 30%, as shown in Figure 8. Kim et al. reported a one-step deposition of Cu_2_O-CuSCN to produce a nanocomposite HTL composed of Cu_2_O nanoparticles (20 nm in size) dispersed in a CuSCN solution with diethyl sulfide [42]. High mobility of Cu_2_O placed at the perovskite/CuSCN interface improved the hole extraction rate and reduced interfacial reaction, improving the PSC efficiencies from 17.7 to 19.2%, and encapsulated devices sustained its PCE over 90% under severe conditions of 85% relative humidity and 85 °C for 720 h, as shown in Figure 9.

### 2.5. Delafossites

Delafossite materials are based on the chemical formula of *AB*O_2_, where *A* is Cu, Pt, Pd, or Ag, and *B* is Al, Ga, Cr, In, Sc, Fe, Y, La, etc. Some common Cu-based delafossite materials are CuAlO_2_, CuCrO_2_, and CuGaO_2_. CuAlO_2_ has a bandgap of 3.75–3.86 eV, valence band of −5.0 to −5.3 eV, and hole mobility of 3.6 cm^2^/Vs [73,74]. *p*-Type CuAlO_2_ has been reported to exhibit decent thermal, chemical, and ambient stability and optical transparency and contains non-toxic and cheap, easily accessible elements. Inserting CuAlO_2_ deposited by direct current (DC) magnetron sputtering on top of ITO and below PEDOT:PSS in a *p*-*i*-*n* configuration resulted in a higher PCE of 14.5% compared to the PCE of the reference device (11.1%) [43]. By inserting 15 nm of CuAlO_2_, the stability of the device improved by maintaining 80% of its initial PCE after storage in ambient conditions for 240 h, whereas the reference device only retained 35% of its initial PCE.

**Figure 10:**
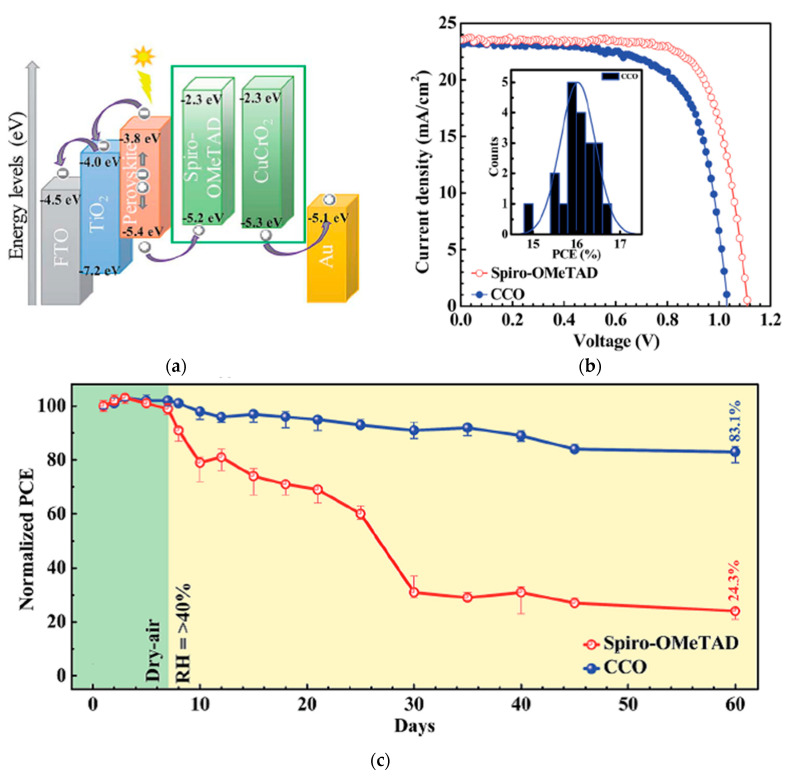
CuCrO_2_-incorporated PSC: (**a**) Schematic illustration of the device architecture and band energy levels. (**b**) Illuminated *J*-*V* scans of the champion cells with various HTLs. (**c**) Efficiency evolution of unencapsulated devices for over 60 days under ambient conditions at room temperature and >40% relative humidity. Reproduced from the work of [12], with permission from the Royal Society of Chemistry, 2018.

**Figure 11:**
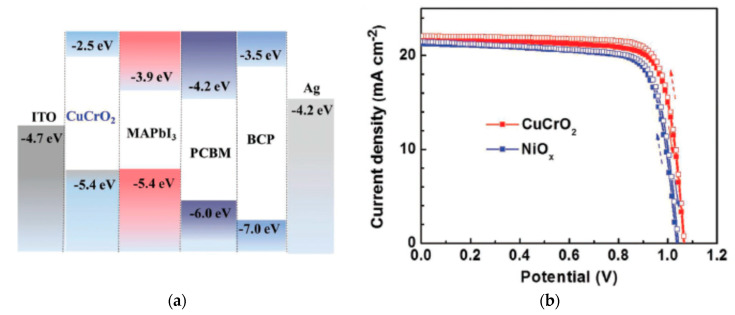

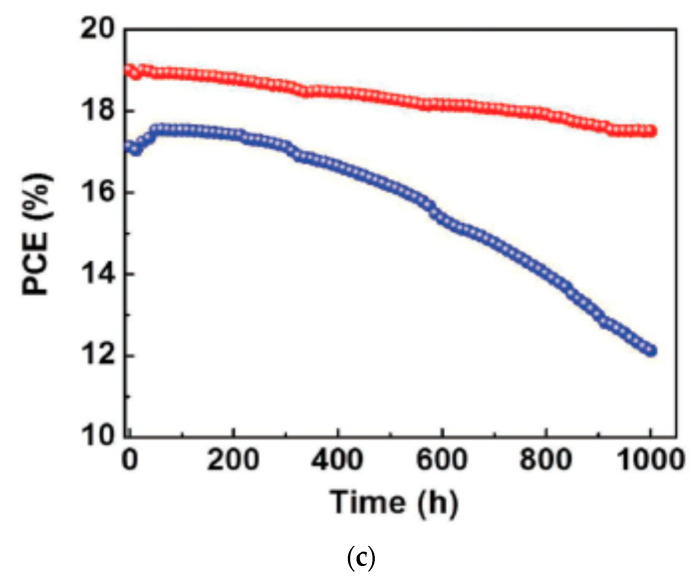
CuCrO_2_-incorporated PSC: (**a**) Energy band diagram with respect to the vacuum level. (**b**) Illuminated *J*-*V* scans of the champion cells with various HTLs. (**c**) Efficiency evolution of unencapsulated devices under 1 SUN in an argon atmosphere. Reproduced from the work of [44], with permission from Wiley, 2018.

CuCrO_2_ has a bandgap of 2.9–3.1 eV while maintaining high transmittance in the wavelength region above 400 nm, valence band energy level of −5.3 eV, and carrier mobility of 0.1–1 cm^2^/Vs with suitable light stability [75]. CuCrO_2_ spin-coated on top of the perovskite layer in an *n*-*i*-*p* structure resulted in a PCE of 16.7%, and retained around 88% of its initial PCE after 500 h under 1 SUN, MPPT in a nitrogen atmosphere at room temperature, as shown in Figure 10 [12]. In a *p*-*i*-*n* structure, applying CuCrO_2_ nanocrystals as the HTL resulted in an efficiency of 19% [44] and retained ~95% of its initial PCE after continuous 1 SUN illumination in argon atmosphere for 1000 h, as shown in Figure 11. Here, CuCrO_2_ nanocrystals function as an HTL as well as a UV-blocking underlayer to improve photostability. CuCrO_2_ has also been doped with Mg, with improved conductivity from 1 to 220 S cm^−1^, resulting in a PCE of 13.1% [45]. The PCE was further improved by Zhang et al. to 14.1% [46].

**Figure 12:**
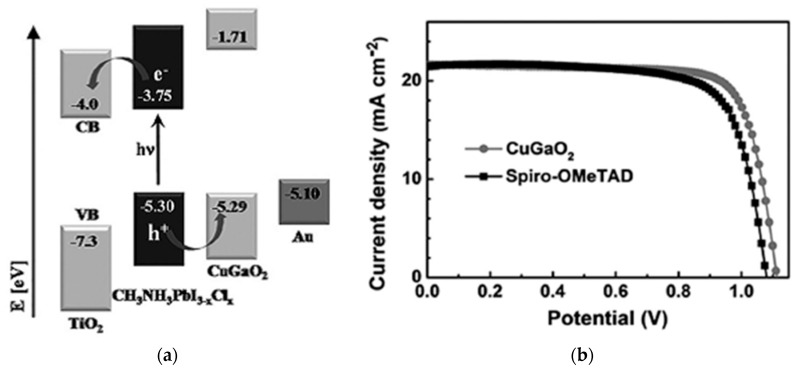

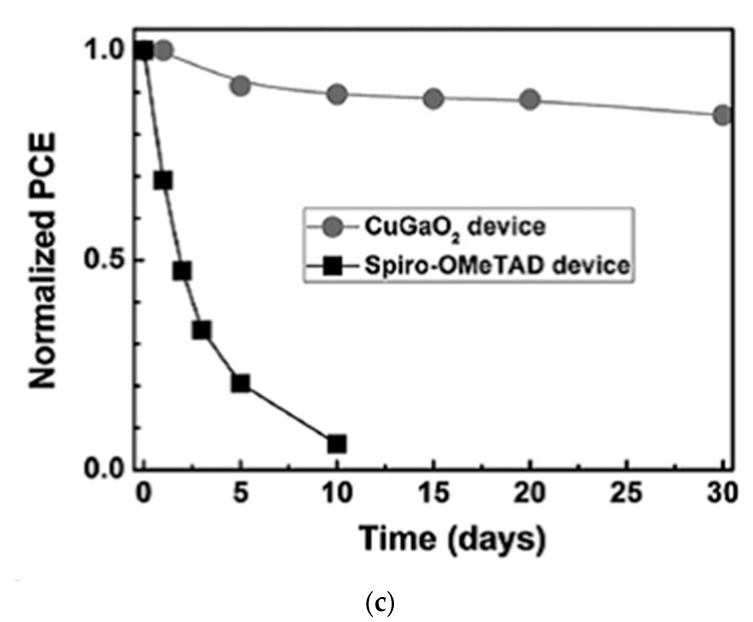
CuGaO_2_-incorporated PSC: (**a**) Energy band diagram with respect to the vacuum level. (**b**) Illuminated *J*-*V* scans of the champion cells with various HTLs. (**c**) Normalized efficiency evolution of unencapsulated devices under ambient atmosphere (25 °C, 30–55% relative humidity). Reproduced from the work of [47], with permission from Wiley, 2017.

**Figure 13:**
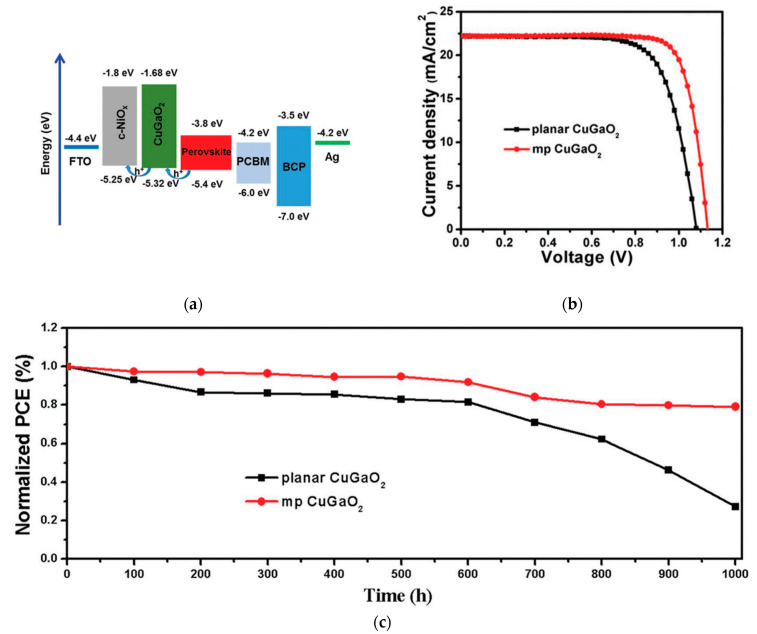
Mesoporous-CuGaO_2_-incorporated PSC: (**a**) Energy band diagram with respect to the vacuum level. (**b**) Illuminated *J*-*V* scans of the champion cells with different HTLs. (**c**) Normalized efficiency evolution of unencapsulated devices at 85 °C in a nitrogen atmosphere. Reproduced from the work of [48], with permission from Wiley, 2018.

CuGaO_2_ has a bandgap of 3.6 eV, a valence band energy level of ~−5.3 eV, and hole mobility of 10^−2^–10^−1^ cm^2^/Vs [76,77,78]. CuGaO_2_ has suitable heat and ambient stability compared to spiro-OMeTAD. CuGaO_2_ spin-coated on top of the perovskite layer in an *n*-*i*-*p* configuration resulted in a PCE of 18.5% and retained over 90% of its initial PCE after storage in ambient air at 25 °C and 30–55% relative humidity for 30 days without encapsulation, which is superior to the spiro-OMeTAD-based PSCs, as shown in Figure 12 [47]. A mesoporous CuGaO_2_ coated on top of NiO_x_ on FTO resulted in a PCE of 20%, which is superior to that of the planar cell (16.7%) [48], and maintained over 80% of its original PCE after 1000 h in a nitrogen atmosphere at 85 °C of unencapsulated devices, as shown in Figure 13. 

**Figure 14:**
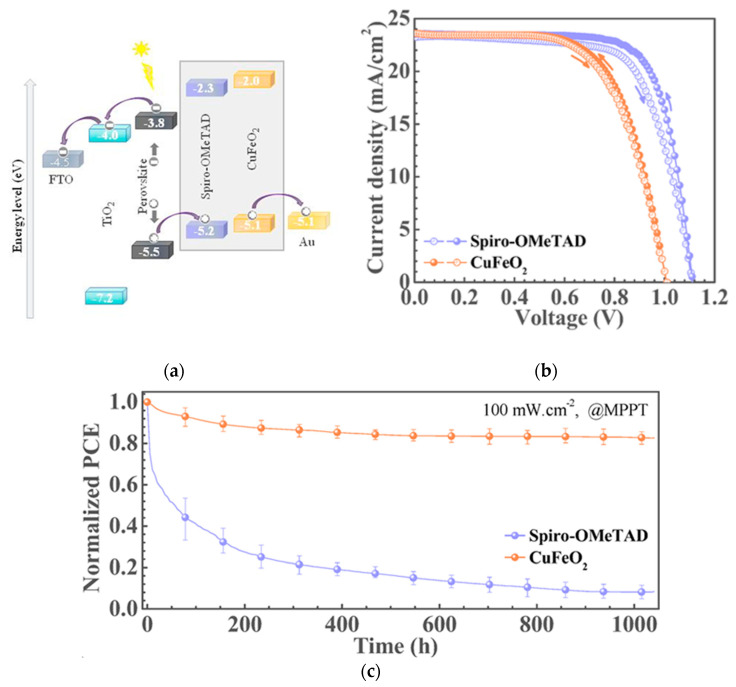

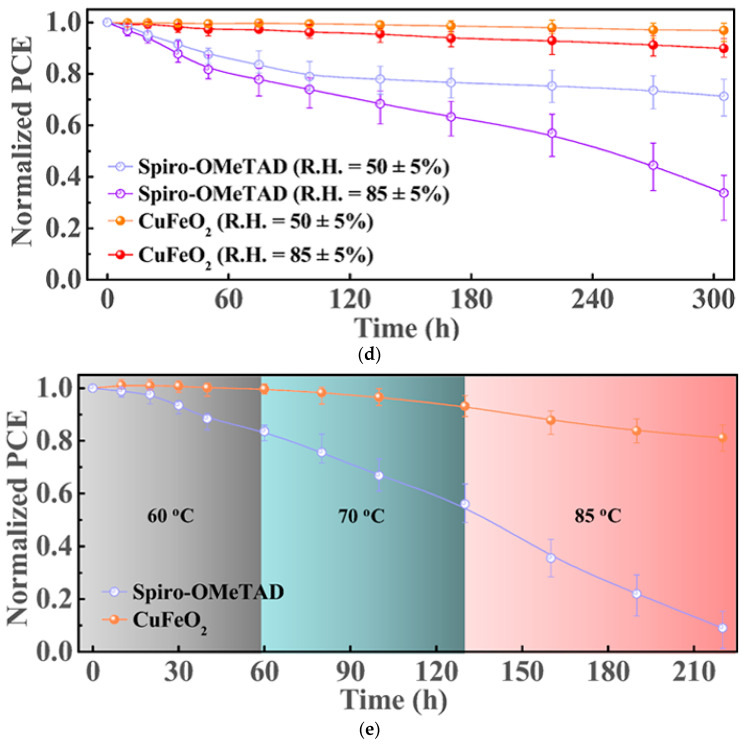
CuFeO_2_-incorporated PSC: (**a**) Energy band diagram with respect to the vacuum level. (**b**) Illuminated *J*-*V* scans of the champion cells with different HTLs. (**c**) Normalized PCE evolution of unencapsulated devices under continuous 1 SUN illumination, MPPT in a nitrogen atmosphere. Normalized PCE decay over time in various (**d**) humidity and (**e**) temperature conditions. Reproduced from the work of [49], with permission from the American Chemical Society, 2019.

CuFeO_2_ is also a cost-effective and highly light, moisture, and thermally stable material for an HTL candidate. PSCs with CuFeO_2_ exhibit suitable thermal, moisture, and photostability compared to PSCs based on spiro-OMeTAD [49]. Unencapsulated devices with CuFeO_2_ retained about 85% of their initial PCE under 1 SUN at MPPT for over 1000 h in nitrogen, whereas spiro-OMeTAD devices dropped to 10%, as shown in Figure 14. Thermal and humidity stability tests show that CuFeO_2_-based devices retained 80% of their initial PCE after exposure to 70 °C for 120 h and retained over 90% of their initial PCE after exposure to 80 ± 5% relative humidity for 300 h. Among the delafossite-based PSCs, devices with CuCrO_2_ and CuGaO_2_ reported high efficiencies above 19%. PSCs with CuCrO_2_ show suitable light stability, while CuGaO_2_-based devices show suitable heat and ambient stability compared to spiro-OMeTAD-based devices.

### 2.6. Copper Sulfide

**Figure 15:**
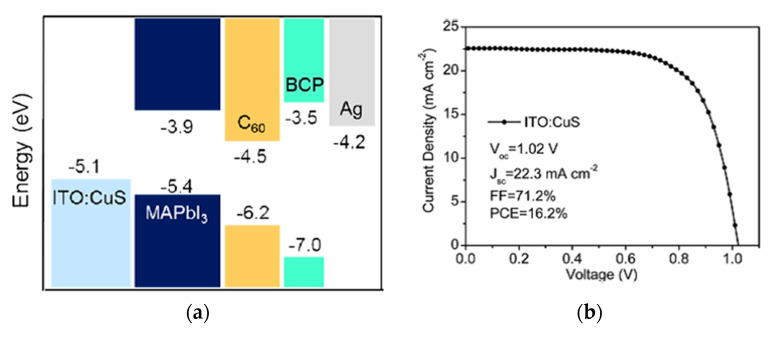

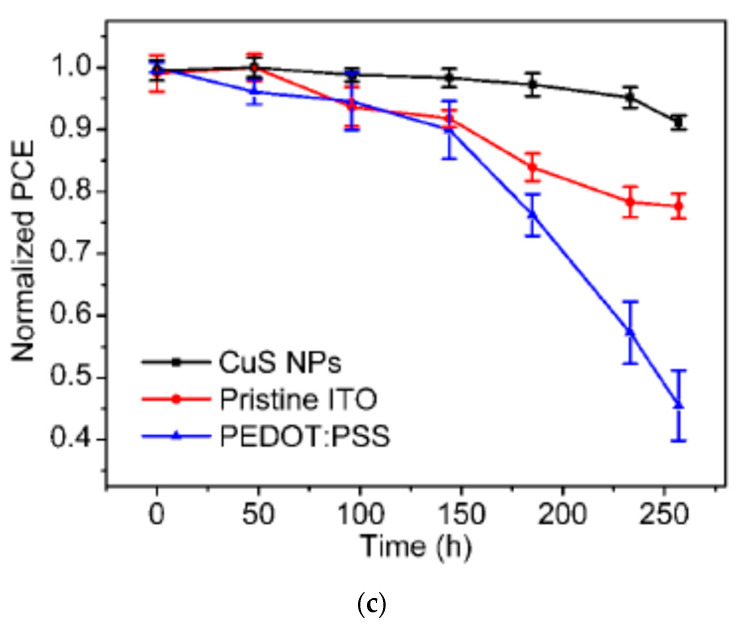
CuS-incorporated PSC: (**a**) Energy band diagram with respect to the vacuum level. (**b**) Illuminated *J*-*V* scan of the champion cell. (**c**) Normalized PCE evolution of unencapsulated devices as a function of storage time in ambient air for PSCs with various HTLs. Reproduced from the work of [50], with permission from the American Chemical Society, 2016.

Copper sulfide (CuS) is a *p*-type semiconductor, which has also been used in the fields of gas sensors, catalysis, and nonlinear optical materials [79,80]. CuS has been investigated to replace PEDOT:PSS in OPV, exhibiting decent performance compared to devices based on PEDOT:PSS [81]. CuS nanoparticles were coated on top of ITO in a *p*-*i*-*n* configuration PSCs, which resulted in a PCE of 16.2% [50], and maintained over 90% of its initial PCE in air without encapsulation for 260 h, shown in Figure 15. CuS nanoparticles can modify the surface of ITO by tuning the surface work function, reducing the interfacial carrier injection barrier, and enabling the hole extraction efficiency between the ITO and perovskite layers, but not ruin the transmittance and surface roughness of ITO.

### 2.7. Cobalt Oxide

**Figure 16:**
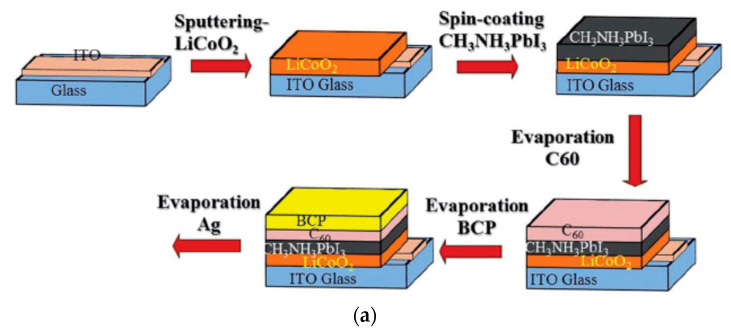

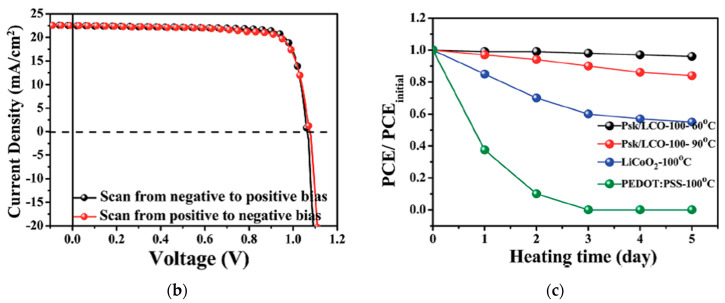
LiCoO_2_-incorporated PSC: (**a**) Schematic illustration of fabrication steps of PSC with LiCoO_2_ as the HTL. (**b**) Illuminated *J*-*V* scans of the champion cell. (**c**) Normalized PCE evolution of unencapsulated devices as a function of storage time with various temperatures in an inert atmosphere. Reproduced from the work of [53], with permission from the Royal Society of Chemistry, 2018.

Cobalt oxide (CoO_x_) has a favorable valence band energy level of −5.3 eV. Co_3_O_4_ applied by screen printing to an *n*-*i*-*p* configuration of a ZrO_2_ scaffold resulted in a PCE of 13.3% for carbon-based PSCs [52]. CoO_x_ spin-coated on top of ITO in a *p*-*i*-*n* configuration resulted in a PCE of 14.5% [51]. Shalan et al. reported that according to photoluminescence decays of perovskite deposited on various HTLs, CoO_x_ had a faster hole-extracting time of 2.8 ns compared to PEDOT:PSS (17.5 ns) and NiO_x_ (22.8 ns). CoO_x_-based PSC retained 90% of its initial PCE after storage in a nitrogen atmosphere for 1000 h. Lithium cobalt oxide (LiCoO_2_) prepared by radio frequency (RF) magnetron sputtering in a *p*-*i*-*n* structured device resulted in a PCE of 19.1%, with high efficiency stable up to 90 °C, and 60% of the initial PCE was retained after continuous thermal stress at 100 °C for 5 days in an inert atmosphere, showing higher stability than the PEDOT:PSS-based device (Figure 16) [53]. UV-ozone-treated LiCoO_2_ exhibits a super-hydrophilic surface that can be wetted easily by the perovskite precursor solution and made wetting of a large-area substrate of 10 cm × 10 cm possible.

### 2.8. Chromium Oxide

**Figure 17:**
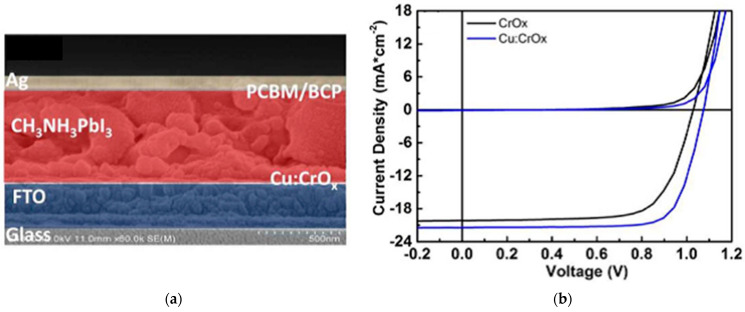

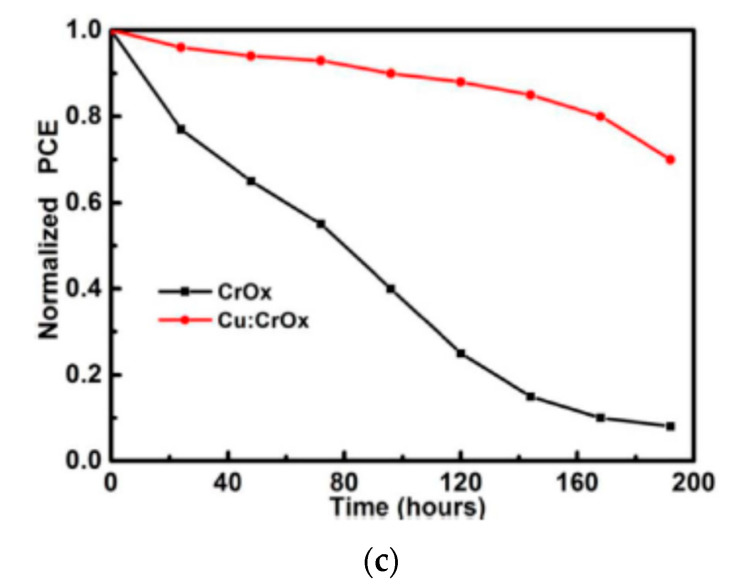
Cu:CrO_x_-incorporated PSC: (**a**) Cross-sectional SEM of PSC device stack. (**b**) *J*-*V* scans of the champion cells with various HTLs. (**c**) Normalized PCE evolution of unencapsulated devices as a function of storage in a dry box with 30% relative humidity at 20 °C. Reproduced from the work of [54], with permission from Elsevier, 2018.

Chromium oxide (CrO_x_) has also been investigated to replace organic HTLs in PSCs. Cu doping of CrO_x_ can suppress the surface hydroxylation and hexavalent chromium ions, which are harmful to the interface stability of PSCs. Cu-doped CrO_x_ in *p*-*i*-*n* PSC devices resulted in a PCE of 17.7%, and maintained over 70% of its original PCE after 190 h in 30% humidity 20 °C without encapsulation, as shown in Figure 17, whereas undoped CrO_x_-based PSCs resulted in a PCE of 14.8% and maintained less than 10% of its initial PCE [54]. PSCs with Cu-doped CrO_x_ shows superior ambient stability than PSCs with undoped CrO_x_.

### 2.9. Molybdenum Oxide

Molybdenum oxide (MoO_3_) is an *n*-type semiconductor with a deep conduction band energy level, making it an appropriate HTL. Due to its suitable energy alignment properties, MoO_3_ has already been used in OPV [82]. Thermally evaporated MoO_x_ on ITO in a *p*-*i*-*n* configuration resulted in a PCE of 13.1% [55]. UV-ozone treatment of MoO_x_ was required to increase the wettability of the perovskite formation process. Titanium-doped MoO_2_ nanoparticles by a scalable solvothermal cracking process applied to an *n*-*i*-*p* PSC configuration resulted in a PCE of 15.8% [56]. Titanium-doping in MoO_2_ nanoparticles produces stronger Mo-O bonding and thus, enhances the stability against humidity. Xie et al. reported that reduced graphene oxide (RGO) doping is an effective method to make MoO_x_ a promising HTL [57]. Conductive MoO_x_:RGO can facilitate perovskite crystallization and reduce the *V_OC_* loss, resulting in a PCE of 18.2% and *V_OC_* of 1.12 V.

### 2.10. Vanadium Oxide

**Figure 18:**
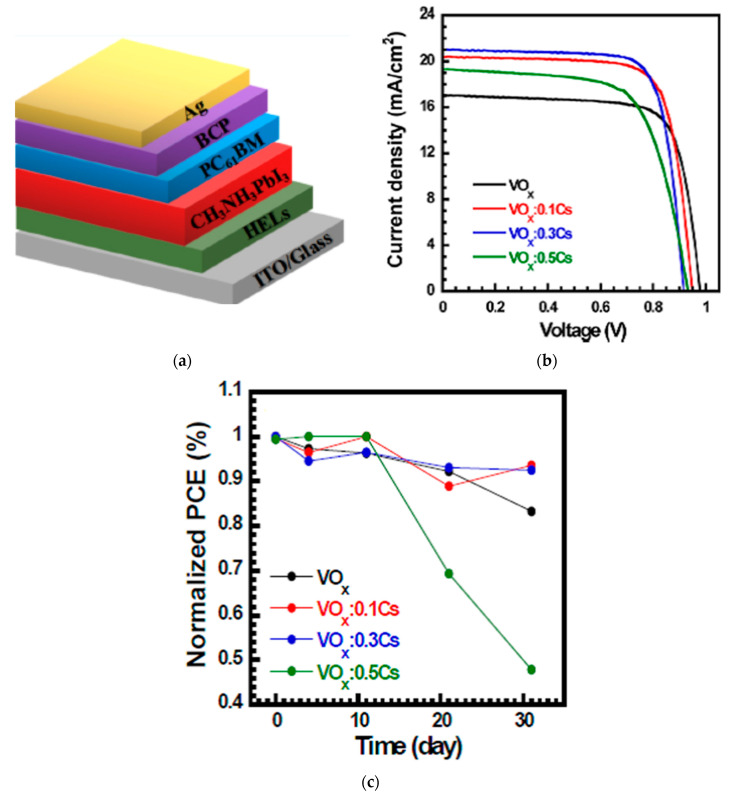
Cs:VO_x_-incorporated PSC: (**a**) Illustration of the PSC device stack. (**b**) Illuminated *J*-*V* scans of the champion cells with various HTLs. (**c**) Normalized PCE evolution as a function of storage time in ambient air with 10–20% relative humidity at 25–30 °C for devices without encapsulation. Reproduced from the work of [59] (https://pubs.acs.org/doi/abs/10.1021/acsomega.7b01944 accessed on 15 December 2021), with permission from the American Chemical Society, 2018.

Vanadium oxide (VO_2_) is also an *n*-type semiconductor with a deep conduction band energy level, making it an appropriate HTL [82,83]. VO_x_ on top of the perovskite layer using a ZrO_2_ scaffold resulted in a PCE of 15.8% [58]. VO_x_ was applied by post-treatment of the perovskite/carbon interface to facilitate the charge transfer from the high work function of VO_x_ while not sacrificing the conductivity of carbon. A low-temperature solution-processed Cs-doped VO_x_ was applied on top of ITO in a *p*-*i*-*n* structure resulted in a PCE of 14.5% [59], and maintained 94% of its initial PCE value after 720 h in the air (50–70% humidity) without encapsulation, showing suitable ambient stability, as shown in Figure 18. Introducing Cs to VO_x_ improved the electrical conductivity and can change the phase separation pattern and microstructural film morphology. The enlarged surface roughness resulted in enhanced interfacial adhesion between the HTL and perovskite layer.

## 3. Conclusions

In summary, the recent progress of inorganic HTL-based PSCs and the roles of the inorganic HTLs on device performance and stability against heat, humidity, bias, and light are discussed. The efficiencies of inorganic HTL-based PSCs reported so far are over 20% for Cu:NiO_x_, Li,Mg:NiO_x_, CuSCN, CuI-CuSCN, and CuGaO_2_, which is lower but still approaching the efficiencies for organic HTL-based PSCs. Superior device stability of inorganic HTL-based PSCs to organic HTL-based PSCs has been reported, showing the potential of inorganic HTLs to replace organic HTLs in PSC devices. Further investigation on increasing the PCEs of inorganic HTL-based PSCs and a better understanding of the degradation and working mechanisms are still required.

The general criteria for selecting potential HTL candidates are also discussed. The valence band energy levels should be close to that of the perovskite layer to facilitate efficient carrier transport and appropriate conduction band energy levels to impede recombination at the HTL/perovskite interface. The carrier mobility should be high to reduce resistance and loss during transport, while the transparency should be high enough to reduce input solar radiation loss. In a *p*-*i*-*n* structure, the nucleation and wettability of the perovskite solution on the HTL surface become important. In an *n*-*i*-*p* structure, the stability of the HTL becomes important because of its contact with humidity and oxygen.

Although this review focused on single-junction unit-cell perovskite solar cells, large-area coating methods and tandem configurations, which consist of wide-bandgap perovskite solar cells on top of lower bandgap materials, such as silicon, Cu(In,Ga)Se_2_, and tin-related materials [15,84,85,86,87,88], need to be considered for commercialization [89]. Thus, inorganic HTL incorporated into tandem perovskite solar cells and coating methods for large-area devices are future directions to be taken. There may be temperature or fabrication limitations of the inorganic HTL in these tandem configurations, depending on the bottom cell. Especially, flexible tandem solar cells will have a limit on the processing temperatures of the layers. Obtaining highly uniform pinhole-free films of the inorganic HTL over a large area will also be important to consider for commercialization.

## Figures and Tables

**Table 1:** Summary of inorganic HTL-based perovskite solar cells.

Material	Method	Structure	Device Stack	*J_SC_* (mA/cm^2^)	*V_OC_* (V)	*FF* (%)	*η* (%)	Institute, Year [Ref]
NiO	Sputtering	*n*-*i*-*p*	FTO/TiO_2_/MAPbI_3−x_Cl_x_/NiO_x_/Ni	17.9	0.77	53.0	7.3	TMU, 2015 [17]
NiO	Spin Coating	*n*-*i*-*p*	FTO/bl-TiO_2_/mp-TiO_2_/MAPbI_3−x_Cl_x_/NiO_x_/Au	19.5	0.88	53.1	9.1	Peking, 2017 [18]
NiO	PLD	*p*-*i*-*n*	ITO/NiO_x_/MAPbI_3_/PCBM/LiF/Al	20.2	1.06	81.3	17.3	KRICT, 2015 [19]
NiO	Solution Process	*p*-*i*-*n*	ITO/NiO_x_/MAPbI_3_/C_60_/Bis-C_60_/Ag	21.8	1.03	78.4	17.6	Hong Kong UST, UW, 2016 [20]
Li,Mg:NiO_x_	Spray Coating	*p*-*i*-*n*	FTO/Li,Mg:NiO_x_/FAPbI_3_/PCBM/TiO_x_/Ag	23.1	1.10	81.4	20.7	NIMS, 2017 [21]
NiO	Spatial ALD	*p*-*i*-*n*	ITO/NiO_x_/FA_0.2_MA_0.8_PbI_3_/PC_61_BM/Al	23.0	1.08	81.0	17.1	Cambridge, 2018 [22]
NiO	ALD	*p*-*i*-*n*	FTO/NiO/Cs_0.05_MA_0.95_PbI_3/_PCBM/BCP/AZO/Ag/Al_2_O_3_	22.5	1.03	80.8	18.8	SKKU, 2018 [23]
NiO	PEALD	*p*-*i*-*n*	ITO/NiO/Cs_0.05_(FA_0.83_MA_0.17_)Pb(I_0.83_Br_0.17_)_3_/C_60_/BCP/Cu	21.8	1.07	73.4	17.1	Eindhoven, 2019 [24]
Cu:NiO_x_	Combustion sol-gel	*p*-*i*-*n*	ITO/Cu:NiO_x_/MAPbI_3_/Bis-C_60_/C_60_/Ag	22.2	1.05	76.0	17.7	UW, 2015 [25]
Cu:NiO_x_	Spin Coating	*p*-*i*-*n*	FTO/Cu:NiO_x_/MAPbI_3−x_Cl_x_/PC_61_BM/ZrAcac/Al	23.7	1.12	77.1	20.1	CAS, 2017 [26]
Li_0.05_Mg_0.15_Ni_0.8_O	Spray Pyrolysis	*p*-*i*-*n*	FTO/Li_0.05_Mg_0.15_Ni_0.8_O/MAPbI_3_/Ti(Nb)O_x_/Ag	22.8	1.11	77.1	19.6	NIMS, 2017 [27]
Li:NiO_x_	Spin Coating	*p*-*i*-*n*	ITO/Li:NiO_x_/MAPbI_3−x_Cl_x_/PCBM/Al	21.8	1.12	73.6	18.0	Los Alamos, 2018 [28]
Cs:NiO_x_	Spin Coating	*p*-*i*-*n*	FTO/Cs:NiO_x_/MAPbI_3_/PCBM/ZrAcac/Ag	21.8	1.12	79.3	19.4	Southern UST, 2017 [29]
Mg:NiO_x_	Sputtering	*p*-*i*-*n*	ITO/Mg:NiO_x_/MAPbI_3_/PCBM/ZnMgO/Al	21.3	1.08	79.0	18.2	Hong Kong UST,2017 [30]
Co:NiO_x_	Spin Coating	*p*-*i*-*n*	FTO/Co:NiO_x_/MAPbI_3_/PCBM/Ag	20.5	1.09	79.8	17.8	NTU, 2020 [31]
CuSCN	Doctor blading	*n*-*i*-*p*	FTO/*bl*-TiO_2_/*mp*-TiO_2_/(FAPbI_3_)_0.85_(MAPbBr_3_)_0.15_/CuSCN/Au	21.8	1.10	69.0	16.6	EPFL, 2016 [32]
CuSCN	Spin Coating	*n*-*i*-*p*	FTO/*bl*-TiO_2_/*mp*-TiO_2_/(FAPbI_3_)_0.85_(MAPbBr_3_)_0.15_/CuSCN/Au	23.1	1.04	75.3	18.0	UNIST, 2016 [33]
CuSCN	Spin Coating	*n*-*i*-*p*	FTO/*bl*-TiO_2_/*mp*-TiO_2_/CsFAMAPbI_3−x_Br_x_/CuSCN/rGO/Au	23.4	1.14	77.5	20.4	EPFL, 2017 [34]
CuI	Rapid Doctor Blading	*n*-*i*-*p*	FTO/*bl*-TiO_2_/MAPbI_3_/CuI/Graphite	16.7	0.78	57.0	7.5	Monash, 2015 [35]
CuI	Spin Coating	*p*-*i*-*n*	ITO/CuI/MAPbI_3_/C_60_/BCP/Ag	22.8	1.01	73.0	16.8	Peking, 2016 [36]
CuI-CuSCN	Electrodeposition	*p*-*i*-*n*	ITO/CuI/CuSCN/MAPbI_3_/PC_61_BM/C	20.3	1.10	78.0	20.4	Allagappa, 2021 [37]
CuO_x_	Spin Coating	*p*-*i*-*n*	ITO/CuO_x_/MAPbI_3_/C_60_/BCP/Ag	23.2	0.99	74.4	17.1	Peking 2016 [38]
CuO_x_	Spin Coating	*p*-*i*-*n*	FTO/CuO_x_/MAPbI_3−x_Cl_x_/PCBM/C_60_/BCP/Ag	22.5	1.11	75.8	19.0	Peking, 2016 [39]
CuO_x_	Spin Coating	*p*-*i*-*n*	ITO/CuO_x_/MAPbI_3_/PC_61_BM/ZnO/Al	22.4	1.03	76.0	17.4	Zhejiang, 2017 [40]
Cu_2_O QD	Spin Coating	*n*-*i*-*p*	FTO/bl-TiO_2_/Cs_0.05_FA_0.81_MA_0.14_PbI_2.55_Br_0.45_/Cu_2_O/Au	22.2	1.15	74.2	18.9	Southern UST, 2019 [41]
Cu_2_O-CuSCN	Spin Coating	*n*-*i*-*p*	ITO/SnO_2_/Cs_0.05_(FA_0.85_MA_0.15_)_0.95_PbI_2.55_Br_0.45_/Cu_2_O-CuSCN/Au	23.2	1.05	78.4	19.2	SNU, 2020 [42]
CuAlO_2_	Sputtering	*p*-*i*-*n*	ITO/CuAlO_2/_PEDOT:PSS/MAPbI_3−x_Cl_x_/PCBM/Ag	22.0	0.88	75.0	14.5	Soochow, 2016 [43]
CuCrO_2_	Spin Coating	*p*-*i*-*n*	ITO/CuCrO_2_/MAPbI_3_/PCBM/BCP/Ag	21.9	1.07	81.0	19.0	City Univ. Hong Kong, 2018 [44]
CuCrO_2_	Spin Coating	*n*-*i*-*p*	FTO/*bl*-TiO_2_/*mp*-TiO_2_/Cs_0.05_(MA_0.15_FA_0.85_)_0.95_Pb(I_0.85_Br_0.15_)_3_/CuCrO_2_/ Au	23.2	1.04	69.0	16.7	Mehmetbey, 2018 [12]
Mg: CuCrO_2_	Spin Coating	*p*-*i*-*n*	FTO/Mg: CuCrO_2_/MAPbI_3_/PCBM/BCP/Ag	18.4	1.00	71.3	13.1	SKKU, 2018 [45]
Mg: CuCrO_2_	Spin Coating	*p*-*i*-*n*	ITO/Mg: CuCrO_2_/MAPbI_3_/C_60_/BCP/Ag	19.4	1.01	71.9	14.1	UT Dallas, 2019 [46]
CuGaO_2_	Spin Coating	*n*-*i*-*p*	FTO/*bl*-TiO_2_/MAPbI_3−x_Cl_x_/CuGaO_2_/Au	21.7	1.11	77.0	18.5	UW, 2017 [47]
CuGaO_2_	Spin Coating	*p*-*i*-*n*	FTO/NiO_x_/*mp*-CuGaO_2_/CsFAPb(I,Br)_3_/PC_61_BM/BCP/Ag	22.2	1.13	80.0	20.0	Shanxi Normal Univ., 2018 [48]
CuFeO_2_	Spin Coating	*n*-*i*-*p*	FTO/*bl*-TiO_2_/*mp*-TiO_2_/CsFAMA/CuFeO_2_/Au	23.6	1.01	65.0	15.6	KMU, 2019 [49]
CuS	Spin Coating	*p*-*i*-*n*	ITO/CuS/MAPbI_3_/C_60_/BCP/Ag	22.3	1.02	71.2	16.2	BNL, 2016 [50]
CoO_x_	Spin Coating	*p*-*i*-*n*	ITO/CoO_x_/MAPbI_3_/PCBM/Ag	20.3	0.95	75.5	14.5	Hokkaido Univ., 2016 [51]
Co_3_O_4_	Screen Printing	*n*-*i*-*p*	FTO/*bl*-TiO_2_/*mp*-TiO_2_/ZrO_2_/MAPbI_3_/Co_3_O_4_/C	23.4	0.88	64.0	13.3	NTU, 2018 [52]
LiCoO_2_	Sputtering	*p*-*i*-*n*	ITO/LiCoO_2_/MAPbI_3_/C_60_/BCP/Ag	22.5	1.06	80.0	19.1	NCU, 2018 [53]
Cu:CrO_x_	Spin Coating	*p*-*i*-*n*	FTO/Cu:CrO_x_/MAPbI_3_/PCBM/BCP/Ag	21.4	1.08	76.0	17.7	Wuhan Univ., 2018 [54]
MoO_x_	Thermal Evaporation	*p*-*i*-*n*	ITO/MoO_3_/MAPbI_3_/PCBM/Ag	18.8	0.99	71.0	13.1	NTUT, 2016 [55]
Ti:MoO_2_	Spin Coating	*n*-*i*-*p*	FTO/*bl*-TiO_2_/*mp*-TiO_2_/MAPbI_3_/Ti:MoO_2/_Au	20.1	1.02	77.3	15.8	Kyung Hee Univ., 2017 [56]
MoO_x_:RGO	Spin Coating	*p*-*i*-*n*	ITO/MoO_x_:RGO/MAPbI_3_/PCBM/BCP/Ag	21.0	1.12	77.0	18.2	NU, 2020 [57]
VO_x_	Post-Treatment	*n*-*i*-*p*	FTO/*bl*-TiO_2_/*mp*-TiO_2_/ZrO_2_/MAPbI_3_/VO_x_/C	24.2	0.95	68.5	15.8	Huazhong UST, 2019 [58]
Cs:VO_x_	Spun-Cast	*p*-*i*-*n*	ITO/Cs:VO_x_/MAPbI_3_/PC_61_BM/BCP/Ag	20.7	0.92	76.5	14.5	South China Univ. Tech., 2018 [59]

**Table 2:** Summary of stability of inorganic HTL-based perovskite solar cells.

HTL	Device Stack	Encapsulated	Conditions	Continuous 1 SUN Illumination?	Duration	*η* Maintained	Institute, Year [Ref]
NiO_x_	FTO/NiO_x_/MAPbI_3_/PCBM/CNT:PEI	Y	60 °C, 60%	N	500 h	85%	Tsinghua, 2018 [60]
NiO_x_	ITO/NiO/Cs_0.17_FA_0.83_Pb(Br_0.17_I_0.83_)_3_/LiF/PC_60_BM/SnO_2_/ZnSnO_x_/ITO/LiF/Ag	N	35 °C, 40%, MPPT	Y	1000 h	100%	Stanford, 2017 [61]
NiO_x_	FTO/NiO_x_/Cs_0.05_MA_0.95_PbI_3_/PCBM/BCP/ ALD-AZO/Ag	Y	RT, 20–60%,/85 °C, MPPT	Y	500 h	99.5/87%	SKKU, 2018 [23]
Li,Mg:NiO_x_	FTO/Li,Mg:NiO_x_/FAPbI_3_/PCBM/TiO_x_/Ag	Y	RT, MPPT	Y	500 h	>85%	NIMS, 2017 [21]
CuSCN	FTO/*bl*-TiO_2_/*mp*-TiO_2_/CsFAMAPbI_3–x_Br_x_/CuSCN/rGO/Au	N	60 °C, N_2_, MPPT	Y	1000 h	>95%	EPFL, 2017 [34]
CuI	ITO/CuI/MAPbI_3_/C_60_/BCP/Ag	N	25%, RT	N	300 h	93%	Peking, 2016 [36]
CuO_x_	ITO/CuO_x_/MAPbI_3_/C_60_/BCP/Ag	N	Air	N	200 h	~90%	Peking, 2016 [38]
Cu_2_O QD	FTO/*bl*-TiO_2_/Cs_0.05_FA_0.81_MA_0.14_PbI_2.55_Br_0.45_/Cu_2_O/Au	N	30%, Air	N	720 h	>90%	Southern UST, 2019 [41]
Cu_2_O-CuSCN	ITO/SnO_2_/Cs_0.05_(FA_0.85_MA_0.15_)_0.95_PbI_2.55_Br_0.45_/Cu_2_O-CuSCN/Au	Y	85 °C, 85%	N	720 h	>90%	SNU, 2020 [42]
CuCrO_2_	ITO/CuCrO_2_/MAPbI_3_/PCBM/BCP/Ag	N	Ar	Y	1000 h	~95%	City Univ. Hong Kong, 2018 [44]
CuCrO_2_	FTO/*bl*-TiO_2_/*mp*-TiO_2_/Cs_0.05_(MA_0.15_FA_0.85_)_0.95_Pb(I_0.85_Br_0.15_)_3_/ CuCrO_2_/ Au	N	RT, N_2_, MPPT	Y	500 h	88%	Mehmetbey, 2018 [12]
CuGaO_2_	FTO/NiO_x_/*mp*-CuGaO_2_/CsFAPb(I,Br)_3_/PC_61_BM/BCP/Ag	N	85 °C, N_2_,	N	1000 h	>80%	Shanxi Normal Univ., 2018 [48]
CuGaO_2_	FTO/*bl*-TiO_2_/MAPbI_3−x_Cl_x_/CuGaO_2_/Au	N	25 °C, 30–55%,	N	720 h	>90%	UW, 2017 [47]
CuFeO_2_	FTO/*bl*-TiO_2_/*mp*-TiO_2_/CsFAMA/CuFeO_2_/Au	N	N_2_, MPPT	Y	1000 h	85%	KMU, 2019 [49]
CuS	ITO/CuS/MAPbI_3_/C_60_/BCP/Ag	N	Air	N	260 h	>90%	BNL, 2016 [50]
LiCoO_2_	ITO/LiCoO_2_/MAPbI_3_/C_60_/BCP/Ag	N	90 °C, Inert Atmosphere	N	120 h	>90%	NCU, 2018 [53]
Cu:CrO_x_	FTO/Cu:CrO_x_/MAPbI_3_/PCBM/BCP/Ag	N	20 °C, 30%,	N	190 h	>70%	Wuhan Univ., 2018 [54]
Cs:VO_x_	ITO/Cs:VO_x_/MAPbI_3_/PC_61_BM/BCP/Ag	N	RT, 50–70%, Air	N	720 h	94%	South China Univ. Tech., 2018 [59]

## Data Availability

Data sharing not applicable.
